# Technical Note on Simplified Free Gingival Graft Using Tack Fixation (sFGG)

**DOI:** 10.3390/medicina59122062

**Published:** 2023-11-22

**Authors:** Won-Pyo Lee, Jae-Seek You, Ji-Su Oh

**Affiliations:** 1 Department of Periodontology, School of Dentistry, Chosun University, Gwangju 61452, Republic of Korea; 2 Department of Oral and Maxillofacial Surgery, School of Dentistry, Chosun University, Gwangju 61452, Republic of Korea; applit375@chosun.ac.kr (J.-S.Y.); jsoh@chosun.ac.kr (J.-S.O.)

**Keywords:** dental implants, mouth mucosa, oral surgical procedures, peri implantitis, vestibuloplasty

## Abstract

Free gingival graft (FGG) is the gold standard procedure for the reliable augmentation of lost keratinized mucosa (KM) around dental implants. This conventional surgical approach has its drawbacks, including limitations in manipulation, the requirement for suturing, postoperative discomfort, and pain. This case report aimed to evaluate the efficacy of a simplified free gingival graft (sFGG) in addressing the issue of inadequate keratinized mucosa around dental implants. Fixation tacks were used to perform the sFGG procedure. Initially, a partial-thickness flap was created and apically repositioned. The gingival graft was harvested from the palate with a narrow profile and securely affixed to the recipient site using 5 mm long fixation tacks. Significant gains in keratinized mucosa were achieved and successfully maintained within 1 year. Consequently, the sFGG technique emerges as a simple and reliable treatment approach for managing inadequate keratinized mucosa around dental implants.

## 1. Introduction

At the 2017 World Workshop, jointly organized by the American Society of Periodontology and the European Society of Periodontology, the term “keratinized mucosa” was defined as the span extending from the peri-implant mucosal margin to the movable lining mucosa [1]. However, the necessity of a specific width of keratinized mucosa around dental implants is controversial due to conflicting research findings [2,3,4,5,6]. While some studies have suggested a positive correlation between an adequate width of keratinized mucosa and improved implant survival rates and soft tissue health [2,3,4], others have failed to establish such a connection [5,6]. Notably, insufficient keratinized mucosa can lead to discomfort during toothbrushing [7,8], complicating oral hygiene management and potentially resulting in plaque accumulation [9].

Methods for increasing the width of keratinized mucosa have been investigated, including apically positioned flap (APF) and free gingival graft (FGG) [10]. APF procedure offers the advantage of not requiring a donor site, making it easier to perform than other methods. However, it may exhibit an excessive recurrence tendency. FGG procedure necessitates a donor site but is considered a standard approach for obtaining keratinized mucosa [11]. While the technique of FGG is recognized for its high success and predictability, it comes with inherent drawbacks, including increased patient discomfort, notable postoperative graft shrinkage, and compromised esthetic outcomes [12]. Acknowledging these limitations, alternatives in the form of soft-tissue substitutes have been introduced as viable options to autogenous tissues [13]. A recent meta-analysis suggests that soft-tissue substitutes of xenogeneic origin hold promise as alternatives to autogenous grafts, demonstrating a comparable increase in peri-implant keratinized tissue (KT) width with reduced patient morbidity, shorter surgical time, and superior esthetic outcomes [14]. However, in a recent network meta-analysis using the apically positioned flap as a reference treatment, FGG emerged as the sole surgical approach among other materials (i.e., CGT, collagen matrix, acellular dermal matrix) to yield a significantly greater KT width at the implant sites (1.14 mm; *p*  =  0.02) [15]. Nevertheless, the time-consuming suturing process for FGG fixation in the recipient area and the operator’s skill level can be challenging [16].

This technical note presented a simplified FGG (sFGG) technique, utilizing fixation tacks to achieve sufficient keratinized mucosa around dental implants.

## 2. Case Report

### 2.1. Surgical Procedures of the Simplified Free Gingival Graft Using Fixation Tacks (sFGG)

Procedures and measurements of the sFGG technique were performed by a skilled periodontologist (W.-P.L.). All sFGG cases utilized 5 mm long fixation tacks (Bone tack, Osung, Gimpo, Republic of Korea). The procedure was performed under local anesthesia using 2% Lidocaine HCl (1:80,000 Epinephrine, Yuhan Corporation, Seoul, Republic of Korea) on both the recipient and donor sites to increase patient comfort and cooperation. Before surgery, patients gargled with a 0.1% chlorhexidine solution (Hexamedine solution, Bukwang Pharm. Co., Seoul, Republic of Korea) for 1 min.

The initial step involved creating a partial-thickness flap using a #15c blade (Osung, Gimpo, Republic of Korea), beginning near the mucogingival junction of the peri-implant soft tissue and progressing apically. The flap was carefully shaped to achieve the desired vestibule formation, and absorbable periosteal sutures (Monosyn^®^ 5-0, B.Braun, Melsungen, Germany) were used to secure the apically positioned flap. When the sFGG procedure coincided with the implant secondary surgical procedure, additional steps were undertaken. This included elevating an inner mucoperiosteal flap from the alveolar ridge or performing the punching technique to install the healing abutment to the implant fixture.

Subsequently, a graft with 4–6 mm width and 1.5–2 mm thickness was conventionally harvested from the palatal side. Hemostasis at the donor site was achieved by packing it with cellulose oxide (Surgicel^®^, Johnson & Johnson Medical Korea, Seoul, Republic of Korea). A prefabricated acrylic wafer using a vacuum former (Pro-Vac^®^, Keystone Industries, Gibbstown, NJ, USA) was placed on the palatal side without sutures to enhance patient comfort.

The harvested gingival graft was then secured to the recipient site using fixation tacks at both ends, eliminating the need for sutures. In some cases, a single fixation tack in the center of the graft was used, and simple interrupted absorbable sutures (Monosyn^®^ 5-0, B.Braun, Melsungen, Germany) were added at both ends of the graft. The surgical wound was carefully covered with an absorbable periodontal dressing (Reso-Pac^®^, Hager & Werken GmbH & Co. KG, Duisburg, Germany) to assist in the healing process and protect the surgical wound. A one-week regimen of postoperative analgesia (aceclofenac 100 mg, Dona-A ST, Seoul, Republic of Korea) was prescribed to patients to alleviate postoperative pain and promote recovery. Fixation tacks and acrylic wafers are typically removed one-week postoperatively.

### 2.2. Case

**Figure 1:**
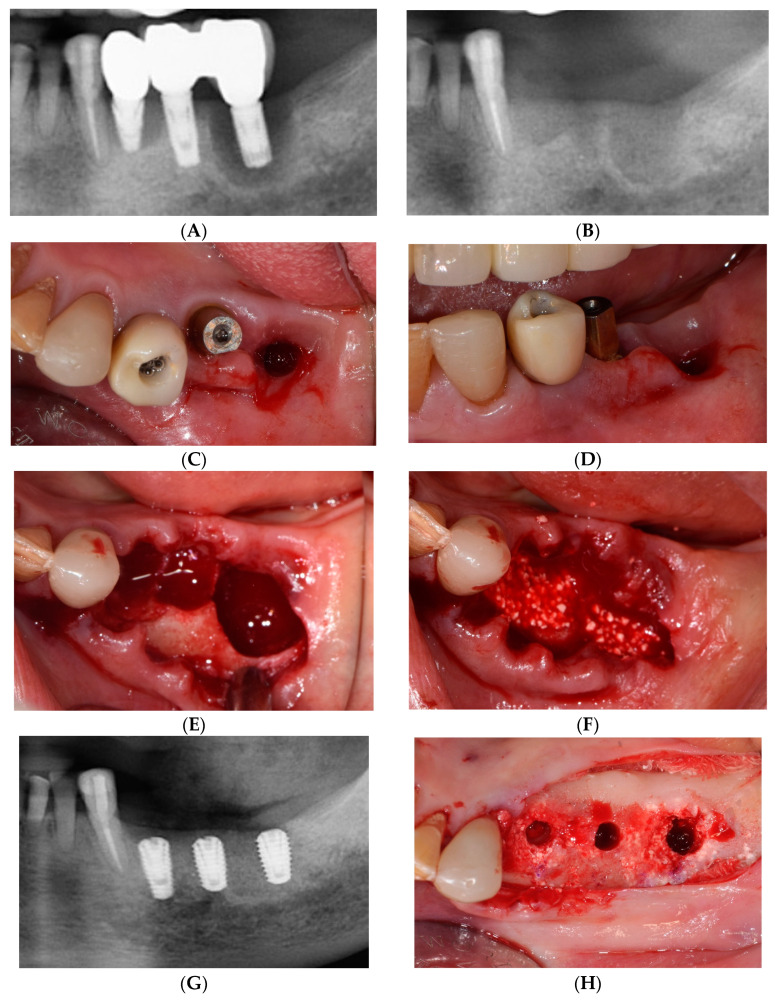
(**A**) Panoramic radiograph at the initial examination. (**B**) Panoramic radiograph after alveolar ridge preservation (ARP). (**C**) Occlusal and (**D**) buccal view at the initial examination. (**E**) Fixture removals. (**F**) Bone grafting for ARP using an octacalcium phosphate-based synthetic bone substitute. (**G**) Panoramic radiograph 4 months after ARP. (**H**) Implant placements.

A 74-year-old female patient presented with complaints of discharge and pain originating from a dental implant located on the left side of her mandible. The patient had experienced significant alveolar bone loss due to peri-implantitis, resulting in the loss of the peri-implant keratinized mucosa. After questioning the patient regarding his medical history, no systemic diseases, such as uncontrolled diabetes, which could affect the healing of hard and soft tissues in the oral cavity, were identified. Furthermore, the patient expressed a desire for the reconstruction of the absent maxillary anterior region and the alleviation of discomfort in the left mandibular posterior region through the use of implant-fixed prostheses. Consequently, with the patient’s informed consent, the problematic implant was removed, and the patient was booked for reimplantation. During the implant removal procedure, an octacalcium phosphate-based synthetic bone substitute (Bontree^®^; HudensBio Co., Gwangju, Republic of Korea) and a resorbable collagen membrane (Remaix™, Matricel GmbH, Herzogenrath, Germany) were used to preserve the alveolar ridge. Significant improvements were observed 4 months after alveolar ridge preservation, and the subsequent implant placement procedure was carried out in sites #34, #35, and #36 (Figure 1).

**Figure 2:**
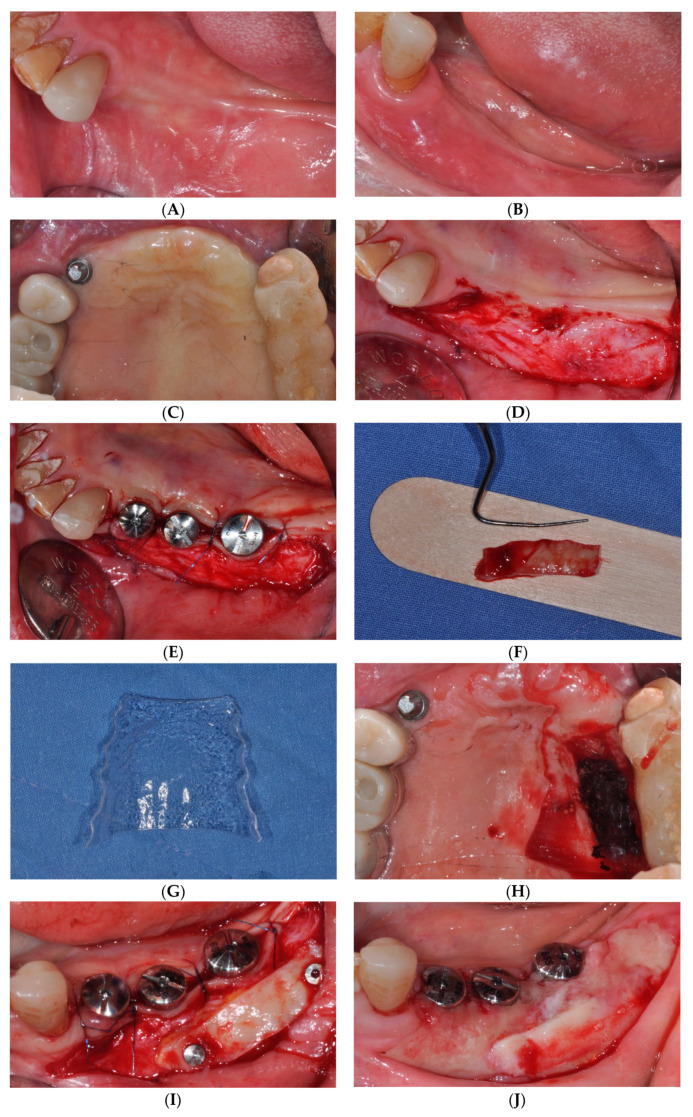

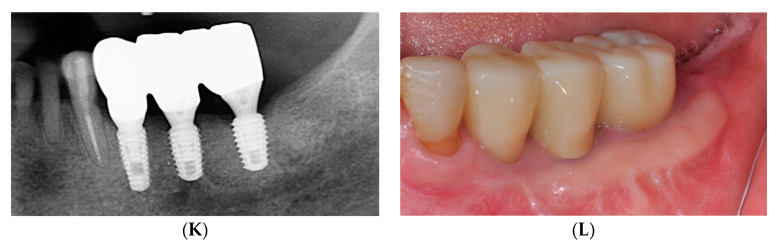
(**A**) Occlusal, (**B**) buccal, and (**C**) palatal view at the initial examination 3 months after implant’s first-stage surgery. (**D**) Creating recipient bed. (**E**) Healing abutment installation. (**F**) The harvested gingival graft. (**G**) A prefabricated acrylic wafer using a vacuum former. (**H**) The acrylic wafer placed at the donor site. (**I**) The gingival graft secured by two fixation tacks. (**J**) One week after simplified free gingival graft (sFGG). (**K**) Panoramic radiograph and (**L**) clinical condition one year after sFGG.

Approximately 3 months following the initial implant surgery, sFGG procedure was performed to augment the previously lost keratinized mucosa during the second implant surgery. A 2 mm thick and 6 mm wide gingival graft was harvested from the palatal side and securely affixed to the recipient site using two fixation tacks to enhance peri-implant soft tissue thickness. The fixation tacks and acrylic wafers were removed 1 week after the surgery. The provisional prosthesis was restored 2 months following the sFGG surgery, while the final prosthesis was completed 4 months post-surgery. This final prosthesis utilized a customized titanium abutment and a zirconia crown. The peri-implant keratinized mucosa was well-maintained at approximately 4 mm for about 1 year following the sFGG procedure (Figure 2).

## 3. Discussion

This case report aimed to evaluate the efficacy of the sFGG procedure in promoting the formation of keratinized mucosa, peri-implant soft tissue thickness, and vestibular depth around dental implants. The sFGG technique simplifies surgery and reduces procedure time to less than that of the conventional FGG procedure, as it requires fewer sutures to secure the gingival graft. Nonetheless, the sFGG procedure achieved and maintained approximately 4 mm of keratinized mucosa during the 12-month follow-up period, similar to the traditional FGG procedure.

Previous studies have shown that the use of APF alone results in minimal keratinized mucosa gain due to high wound contraction and muscle reattachment [10]. Başeğmez et al. reported a gain of 2 mm at 3 months post-surgery with APF, which subsequently decreased to 1.15 mm after 12 months [17]. Lee et al. [16] also observed an approximate 0.5 mm increase in keratinized mucosa width from baseline and a 71% shrinkage rate at 12 months post-surgery with APF. However, combining APF with FGG [13,16,18] or periosteal fenestration techniques [19] resulted in greater gains in keratinized mucosa. Lee et al. [16] reported that the FGG group achieved an increase in keratinized mucosa width of 2.5 mm and a shrinkage rate of approximately 44% after 12 months of healing. Due to its high success rates, predictability, and lack of comparable alternatives, FGG is regarded as the gold standard [16,20].

**Figure 3:**
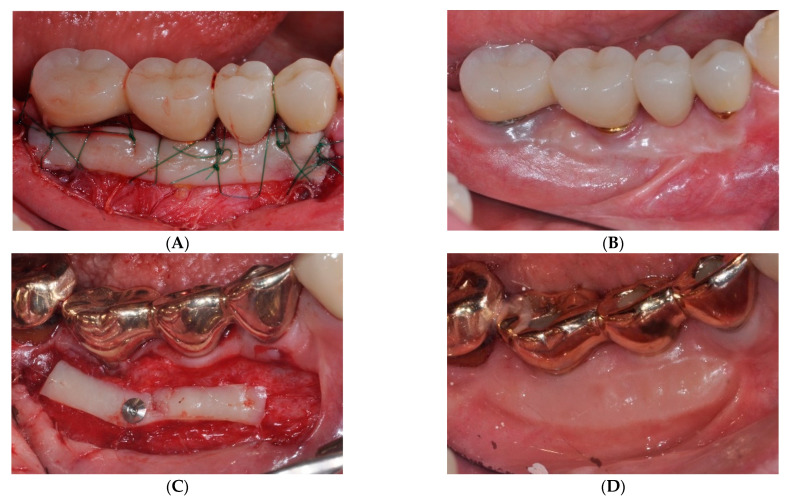
(**A**,**B**) Clinical view at 12 months after traditional free gingival graft (FGG) vs. (**C**,**D**) clinical view at 12 months after simplified FGG. Both methods exhibited a comparable degree of augmentation in keratinized mucosa.

**Figure 4:**
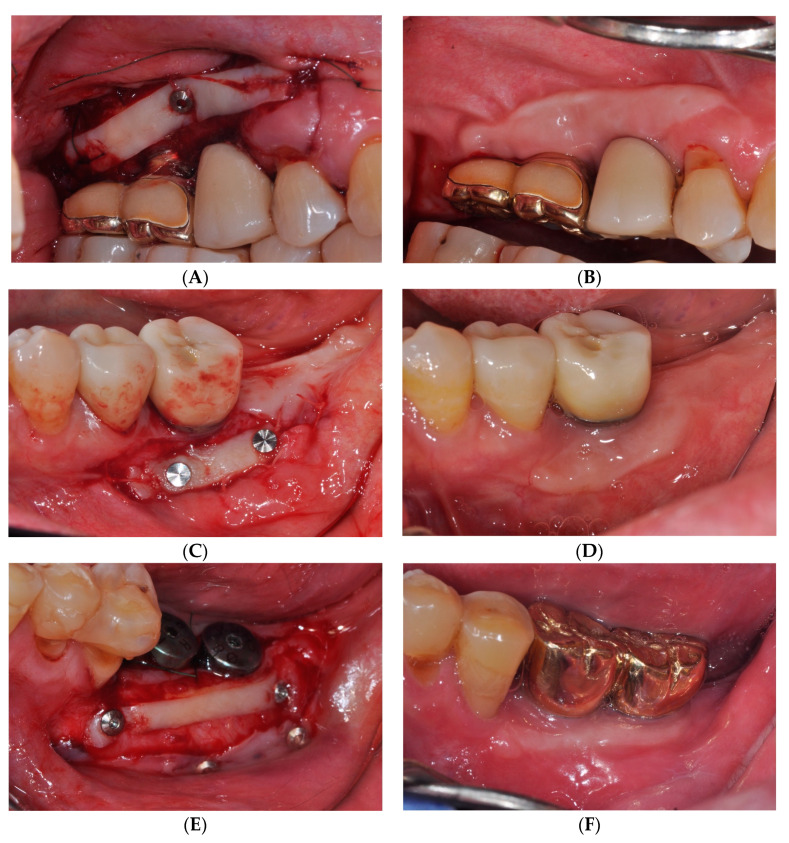
(**A**,**C,E**) Buccal view immediately after performing simplified free gingival graft (sFGG). (**B,D,F**) Clinical view at 12 months after sFGG. Increased keratinized mucosa was observed around the implant even though a narrow width gingival graft was secured.

However, the FGG procedure showed a lower esthetic tissue profile regarding color, contour, and texture than other methods [18]. Urban et al. [21] performed vestibuloplasty by combining a strip gingival graft at the apical part of the recipient bed with a xenogenic collagen matrix at the coronal level to address this issue. The strip gingival graft at the apical part of the recipient bed served as a mechanical barrier against mucosal or muscular reattachment, similar to the role of scar formation in periosteal fenestration surgery [16]. Furthermore, previous reports have demonstrated a shrinkage rate of approximately 25–40% after a conventional FGG procedure [21,22], necessitating a larger graft width to compensate for this shrinkage. However, in the case of sFGG, we obtained similar results despite harvesting a gingival graft with a narrower width, which differs from the traditional FGG method (Figure 3). Given that the gingival graft acts as a physical barrier capable of resisting upward alveolar mucosa or muscle rearrangement [21], fixation tacks allow for an easier attachment of the gingival graft to the apical region of the recipient area than conventional methods, resulting in predictable outcomes with narrower grafts (Figure 4).

**Figure 5:**
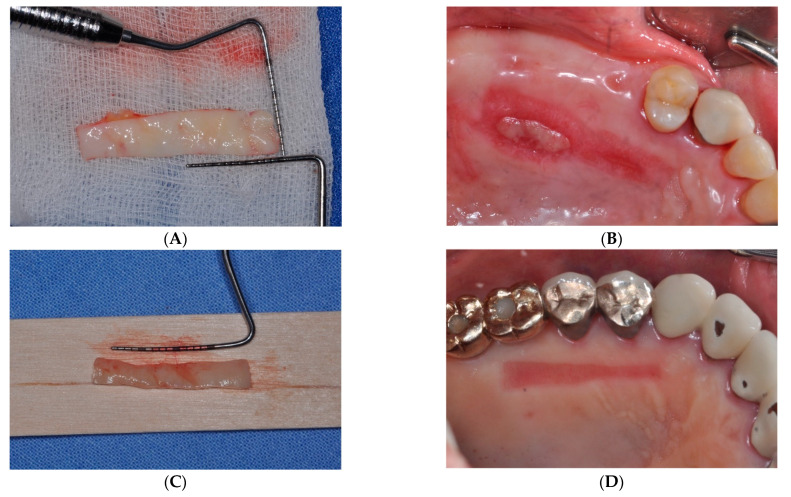
(**A**,**B**) Healing condition at 2 weeks after traditional free gingival graft (FGG) vs. (**C**,**D**) healing condition at 2 weeks after simplified FGG (sFGG). Faster healing was observed in sFGG case, where a narrow width gingival graft was harvested.

One of the drawbacks of the FGG procedure is the discomfort patients experience at the palatal donor site. Various methods have been investigated to address this concern. Morshedzadeh et al. [22] employed a GaAlAs low-power laser and observed faster epithelialization and reduced discomfort compared to procedures without laser application. Doshi et al. [23] tested the topical application of phenytoin on the palatal side, while Femminella et al. [24] achieved similar results by placing a platelet-rich fibrin membrane on the palatal donor site and suturing it to minimize discomfort. The graft is harvested with a narrow width using the sFGG procedure, which may also serve as a means to mitigate patient discomfort compared to the conventional FGG method (Figure 5). Han et al. [25] demonstrated that the healing rate of the donor site is influenced by the width of the surgical site, with faster epithelial cell migration occurring closer to the edge. Therefore, the sFGG technique can alleviate patient discomfort by creating a narrower donor site.

There are limitations to this study. Prospective clinical investigations employing robust methodologies and larger sample sizes are warranted to validate our findings. Additionally, when performing the sFGG procedure, certain precautions should be taken into account. Firstly, the length of the fixation tack used in the sFGG procedure should be at least 5 mm. This is because a 3 mm fixation tack, typically used for securing barrier membranes, may not provide sufficient anchorage in the alveolar bone beneath the recipient site, especially considering that the gingival graft is approximately 2 mm thick. Secondly, while it is generally recommended to harvest a narrow gingival graft of approximately 4 mm in the sFGG procedure and secure it to the apical area of the recipient site using fixation tacks, there are instances where it may be necessary to increase the thickness of the peri-implant marginal soft tissue simultaneously with the augmentation of keratinized mucosa. In such cases, a wider gingival graft should be harvested. Lastly, the significance of microcirculation in soft tissue management cannot be overstated. In the context of the sFGG procedure, systemic factors, notably uncontrolled diabetes mellitus, can exert a profound adverse impact on wound healing. Therefore, it is prudent to engage in consultation with the patient’s diabetologist and to requisition a documentation of the HbA1C level prior to undertaking the surgical intervention.

## 4. Conclusions

We have demonstrated the efficacy of the sFGG procedure with fixation tacks. This innovative technique provides a simple and proactive means of achieving a significant expansion of peri-implant keratinized mucosa. Even with a narrower gingival graft, we could effectively enhance the keratinized mucosa, reducing patient pain. The success of this approach opens up the possibility for it to serve as a viable alternative to traditional FGG procedures. This simplifies the surgical process and underscores its potential to become a valuable addition to the armamentarium of periodontal and implant clinicians seeking to enhance peri-implant soft tissue thickness and keratinized mucosa in a more patient-friendly manner. Further investigations with larger sample sizes and rigorous methodologies will be essential to validate and expand upon our findings.

## Data Availability

The datasets generated or analyzed during the current study are available from the corresponding author upon reasonable request.
